# Optimization of sonochemical degradation of tetracycline in aqueous solution using sono-activated persulfate process

**DOI:** 10.1186/s40201-015-0234-7

**Published:** 2015-11-04

**Authors:** Gholam Hossein Safari, Simin Nasseri, Amir Hossein Mahvi, Kamyar Yaghmaeian, Ramin Nabizadeh, Mahmood Alimohammadi

**Affiliations:** Department of Environmental Health Engineering, School of Public Health, Tehran University of Medical Sciences, Tehran, Iran; Center for Water Quality Research, Institute for Environmental Research, Tehran University of Medical Sciences, Tehran, Iran; Center for Solid Waste Research, Institute for Environmental Research, Tehran University of Medical Sciences, Tehran, Iran; Center for Air Pollution Research, Institute for Environmental Research, Tehran University of Medical Sciences, Tehran, Iran

**Keywords:** Tetracycline degradation, Persulfate, Response surface methodology, Central composite design, Optimization

## Abstract

**Background:**

In this study, a central composite design (CCD) was used for modeling and optimizing the operation parameters such as pH, initial tetracycline and persulfate concentration and reaction time on the tetracycline degradation using sono-activated persulfate process. The effect of temperature, degradation kinetics and mineralization, were also investigated.

**Results:**

The results from CCD indicated that a quadratic model was appropriate to fit the experimental data (*p* < 0.0001) and maximum degradation of 95.01 % was predicted at pH = 10, persulfate concentration = 4 mM, initial tetracycline concentration = 30.05 mg/L, and reaction time = 119.99 min. Analysis of response surface plots revealed a significant positive effect of pH, persulfate concentration and reaction time, a negative effect of tetracycline concentration. The degradation process followed the pseudo-first-order kinetic. The activation energy value of 32.01 kJ/mol was obtained for US/S_2_O_8_^2-^ process. Under the optimum condition, the removal efficiency of COD and TOC reached to 72.8 % and 59.7 %, respectively. The changes of UV–Vis spectra during the process was investigated. The possible degradation pathway of tetracycline based on loses of N-methyl, hydroxyl, and amino groups was proposed.

**Conclusions:**

This study indicated that sono-activated persulfate process was found to be a promising method for the degradation of tetracycline.

## Background

Tetracycline (TC) is extensively used for the prevention and treatment of infectious diseases in human and veterinary medicine and as feed additives for promote growth in agriculture [1, 2]. Because of their extensive usage, their strongly hydrophilic feature, low volatility [2] and relatively long half-life [3], TC antibiotic has been frequently detected in different environmental matrices: surface waters (0.07-1.34 μg/L) [4], soils (86.2-198.7 μg/kg) [5], liquid manures (0.05-5.36 μg/kg) [5] and in 90 % of farm lagoon samples (>3 μg/L) [6]. In addition to environmental contamination, the occurrence of TC in the aquatic environments would also increase antibiotic resistance genes [7]. However, due to the antibacterial nature of TC, they cannot effectively be removed by conventional biological processes [8]. In wastewater treatment plants, the TC removal efficiency varied in the range of 12 % to 80 % [9, 10]. For example, concentrations of TC residues have been detected in values of 0.97 to 2.37 μg/L in the final effluent from wastewater treatment plants [11]. Hence, the effort to develop new processes to minimize the tetracycline residues discharges into the environment is become essential. Physicochemical processes such as membrane filtration and adsorption using activated carbon have been used to removal of TC. These processes are not efficient enough, transfer the pollutant from one phase to another [12, 13]. Advanced oxidation processes (AOPs) such as (O_3_/H_2_O_2_, US/O_3_, UV/O_3_, UV/H_2_O_2_, H_2_O_2_/Fe^2+^, US-TiO_2_ and UV-TiO_2_) have been proposed as very effective alternatives to degrade tetracycline antibiotics. The primary of AOPs is production of hydroxyl radical in water, a much powerful oxidant in the degradation of a wide range of organic pollutants [12–15]. Recently, the application of sulfate radical-based advanced oxidation processes (SR-AOPs) to oxidation of biorefractory organics have attracted great interest [16, 17]. Persulfate (PS, S_2_O_8_^2**−**^) is a powerful and stable oxidizing agent (E_0_ = 2.01 V vs. NHE), which has high aqueous solubility and high stability at room temperature as compared to hydrogen peroxide (H_2_O_2_, E_0_ = 1.77 V vs. NHE) [18, 19].

Sulfate radicles could be produced through the activation of persulfate (PS, S_2_O_8_^2**−**^) with ultraviolet [20], heat [21, 22], microwave [23], sonolysis [24], base [25], granular activated carbon [26], quinones [27], phenols [28], soil minerals [29], radiolysis [30] and transition metals [31, 32]. Sulfate radicals are more effective than hydroxyl radical in the oxidation of organic contaminants. They have higher redox potentials, longer half-life and higher selectivity in the oxidation of organic contaminants (SO_4_^**-•**^, E_0_ = 2.5-3.1, half-life = 30–40 μs) than hydroxyl radical (HO^**•**^, E_0_ = 1.89–2.72 V, half-life = 10^**−3**^ μs) [33–39]. Hence, the organic pollutants could be oxidized entirely by SO_4_^-•^, especially benzene derivatives compounds [18]. Generally, sulfate radical reacts with organic contaminants predominantly through selective electron transfer, while hydroxyl radical mainly reacts through hydrogen abstraction and addition. Therefore, the possibility of sulfate radical scavenging by nontarget compounds is lower than hydroxyl radical [39–42].

Sonochemical treatment is an emerging and efficient process that applied pyrolytic cleavages to degradation of organic compounds [42, 43]. This process is a cleaner and safe technique compared with UV, ozonation, and has the ability of operation under ambient conditions [43, 44]. However, combination of ultrasound with various processes has been detected as an economical and successful alternative for the degradation and mineralization of some recalcitrant organic compounds in aqueous solution [42]. The combination of ultrasound and persulfate (US/S_2_O_8_^2−^) has been effective for the degradation of compounds such as; methyl tert-butyl ether (MTBE) [45], nitric oxide [18], 1,4-dioxane [46], arsenic(III) [44], amoxicillin [47], tetracycline [48] and dinitrotoluenes [24]. In aqueous solutions, acoustic cavitation leading to produce plasma in water and free radicals and other reactive species such as HO^**•**^ and H^**•**^ radicals due to the thermal degradation of water according to Reaction (1) and (2). The HO^**•**^ and H^**•**^ radicals can also react with PS to production of more reactive SO_4_^**-•**^ radicals according to Reactions (3) to (7) [42, 44, 49, 50].

1 $$ {\mathrm{H}}_2\mathrm{O}\ \overset{\left)\right)\left)\right)}{\to }\ {\mathrm{H}}_2\mathrm{O}\ \mathrm{plasma} $$

2 $$ {\mathrm{H}}_2\mathrm{O}\ \overset{\left)\right)\left)\right)}{\to }\ {\mathrm{H}\mathrm{O}}^{\bullet } + {\mathrm{H}}^{\bullet } $$

Where “))))))” refers to ultrasonication.

In the presence of S_2_O_8_^2−^:

3 $$ {\mathrm{S}}_2{\mathrm{O}}_8^{-2} + \overset{\left)\right)\left)\right)}{\to }\ 2{\mathrm{S}\mathrm{O}}_4^{-\bullet } $$

4 $$ {\mathrm{SO}}_4^{-\bullet } + {\mathrm{H}}_2\mathrm{O}\to\ {\mathrm{SO}}_4^{2-} + {\mathrm{H}\mathrm{O}}^{\bullet } + {\mathrm{H}}^{+} $$

5 $$ {\mathrm{S}}_2{\mathrm{O}}_8^{-2}+{\mathrm{HO}}^{\bullet}\to {\mathrm{HSO}}_4^{-}+\kern0.5em {\mathrm{S}\mathrm{O}}_4^{-\bullet }+\frac{1}{2}\ {\mathrm{O}}_2 $$

6 $$ {\mathrm{S}}_2{\mathrm{O}}_8^{-2}+{\mathrm{H}}^{\bullet}\to {\mathrm{H}\mathrm{SO}}_4^{-}+\kern0.5em {\mathrm{S}\mathrm{O}}_4^{-\bullet } $$

7 $$ {\mathrm{S}}_2{\mathrm{O}}_8^{-2} + \overset{\mathrm{pyrolysis}}{\to }\ 2{\mathrm{S}\mathrm{O}}_4^{-\bullet } $$

In aqueous solution, Hydroxyl radicals may be produced via the degradation of persulfate and/or ultrasonic irradiation. Ultrasonic irradiation could also lead to cavitation through the formation, growth and collapse of tiny gas bubbles in the water [51]. Moreover, during US irradiation, the collapse of cavitation bubbles leads to higher temperatures and pressures that produces free radicals and other reactive species and would also increase the number of collisions between free radicals and contaminants [42, 44, 49, 50].

The specific objectives of this study were to optimize the TC degradation in aqueous solution using US/S_2_O_8_^2−^ process. Response surface methodology (RSM) is a reliable statistical technique for developing, improving and optimizing processes and can be used to assess the relative significance of several affecting factors with the least experiments [52–54]. Therefore, an experimental design methodology using RSM and CCD was used to evaluate the effect of operational parameters such as initial TC concentration, initial S_2_O_8_^2−^ concentration, initial pH and reaction time on the sonochemical degradation of tetracycline. In addition, the effect of temperature, degradation kinetics, mineralization, changes of ultraviolet Visible (UV–Vis) spectra and the proposed degradation pathway of TC by the US/S_2_O_8_^2−^ process were investigated. This study as part of a PhD dissertation of the first author was performed at Department of Environmental Health Engineering, School of Public Health, Tehran University of Medical Sciences in 2015.

## Materials and methods

### Materials

Tetracycline hydrochloride [C_22_H_25_N_2_O_8_Cl] (AR, 99 %), was provided from Sigma–Aldrich. Chemical properties of tetracycline hydrochloride are shown in Table 1 [2]. Sodium persulfate (Na_2_S_2_O_8_, 98 %) was provided from Sigma–Aldrich. All other chemicals were of analytical grade and were used without further purification. The water used in all experiments was purified by a Milli- Q system.

**Table 1:** Chemical properties of tetracycline hydrochloride

Molecule	Formula	Molecular weight (g/mol)	Solubility (mol/L)	pK_a1_	pK_a2_	pK_a3_
TC	C_22_H_24_O_8_N_2_.HCl	480.9	0.041	3.2 ± 0.3	7.78 ± 0.05	9.6 ± 0.3

### Procedure

**Fig 1:**
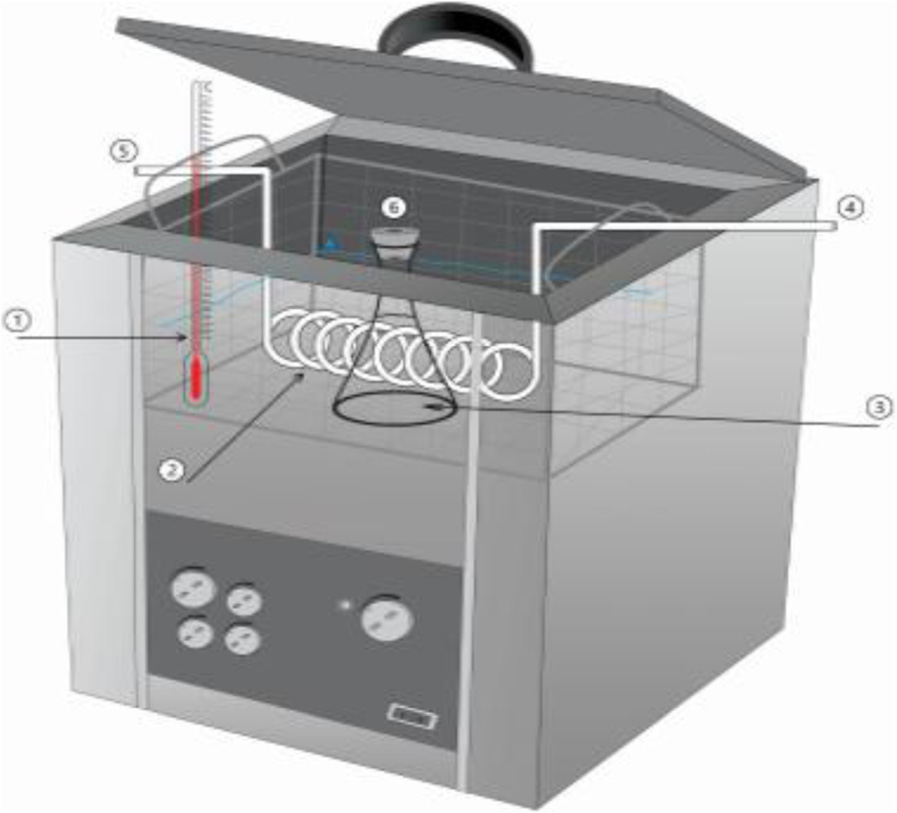
Schematic of the experimental used in this study; (1) temperature controller, (2) water-circulating (3) TC solution reactor (4) cooling water inlet, (5) cooling water outlet (6) sampling port

Schematics of the experimental setup applied in this study is demonstrated in Fig. 1. A stock solution of tetracycline was daily prepared with distilled deionized water and diluted as required initial concentration. Sonochemical treatment was carried out with a fixed volume of 100 mL of TC solution in a glass vessel of 200 mL. The vessel was wrapped with tinfoil in order to avoid any photochemical effects. The pH adjustments were conducted with 1 m NaOH or 1 m HCL (Merck Co.) using a pH meter (E520, Metrohm, Tehran, Iran). Sonochemical treatment was performed with an ultrasonic generator at a frequency of 35 kHz and power of 500 W (Elma, Singen, Germany). The reactor was immersed into the ultrasonic bath and its location was always kept similarly. All experiments were conducted at constant temperature using cooling water and temperature controller. At pre-specified time intervals, 2 mL sample was withdrawn, filtered through 0.22 μm syringe filter and mixed with the same volume of methanol to quench the reaction before analysis [3].

### Analytical methodology

The pH was determined at room temperature using an S-20 pH meter, which was calibrated with pH 4.0 and 7 reference buffer solutions. The concentration of TC in aqueous solution was analyzed by HPLC, with a LC-20 AB pump, Shimadzu, Kyoto, Japan) with a reversed-phase column (VP-ODS-C18 4.6 mm × 250 mm, 5 μm, Shim-Pack, Kyoto, Japan), and UV detector (Shimadzu UV-1600 spectrophotometer). The injection volume was 20 μL; the mobile phase was acetonitrile 0.01 M, oxalic acid solution (31:69, v/v) with a flow rate of 1.0 mL min^−1^. The detection wavelength and retention time of tetracycline were 360 nm and 2.38 min, respectively. In this study, limit of detection (LOD) were found to be 0.02-0.03 mg/L based on linear regression method.

### Experimental design

A central composite statistical experiment design was used to evaluate the effects of four independent variables (initial solution pH (A), initial TC concentration (B), initial S_2_O_8_^−2^ concentration (C) and reaction time (D)) on the TC degradation. The application of RSM provides a mathematical relationship between variables and experimental data can be fitted to an empirical second-order polynomial model as the following Eq. (8). [55–57].

8 $$ Y={\beta}_0+{\beta}_{1\ }A+{\beta}_{2\ }B+{\beta}_{3\ }C+{\beta}_{4\ }D+{\beta}_{12\ } AB+{\beta}_{13\ } AC+{\beta}_{14\ } AD+{\beta}_{23}BC+{\beta}_{24\ }BD + {\beta}_{34}CD+{\beta}_{11\ }{A}^2+{\beta}_{22\ }{B}^2+{\beta}_{33}{C}^2+{\beta}_{44\ }{D}^2 $$

Where, y (%) is the predicted response (TC degradation rate), β_0_ is interception coefficient, β_1,_ β_2,_ β_3_ and β_4_ are the linear coefficients_,_ β_12,_ β_13,_ β_14,_ β_23,_ β_24_ and β_34_ are interaction coefficients, β_11,_ β_22,_ β_33 and_ β_44_ are the quadratic coefficients and A, B, C and D are the independent variables.

The natural and coded levels of independent variables based on the central composite design are shown in Table 2. The experimental values for each independent variables were chosen according to the results obtained from preliminary analysis. Table 3 indicates the four-factor, five-level CCD and the obtained and predicted values for the TC degradation rate (%) using the developed quadratic model. In RSM analysis, the approximation of y was proposed using the fitted second-order polynomial regression model which is called the quadratic model. A quadratic regression is the process of finding the equation of the parabola that fits best for a set of data [58].

**Table 2:** Natural and coded levels of independent variables based on the central composite design

Independent variable	Symbol	Coded levels				
		**−2**	**−1**	**0**	**+1**	+2
		Natural level				
pH	A	2.5	5	7.5	10	12.5
Tetracycline (mg/L)	B	10	30	50	70	90
Persulfate (Mm)	C	1	2	3	4	5
Reaction time (min)	D	30	60	90	120	150

**Table 3:** Four-factor five-level central composite design for RSM

Run	Experimental conditions				TC degradation rate (%)	
	pH (A)	Tetracycline (mg/L) (B)	Persulfate (mM) (C)	Time (min) (D)	Observed (%)	Predicted (%)
1	7.5 (0)	50 (0)	3 (0)	90 (0)	51.06	49.82
2	2.5 (−2)	50 (0)	3 (0)	90 (0)	55.64	55.74
3	7.5 (0)	50 (0)	3 (0)	90 (0)	48.45	49.82
4	5 (−1)	70 (+1)	4 (+1)	60 (−1)	45.16	44.64
5	5 (−1)	30 (−1)	4 (+1)	120 (+1)	86.62	86.33
6	10 (+1)	70 (+1)	2 (−1)	120 (+1)	61.02	61.32
7	7.5 (0)	50 (0)	3 (0)	150 (+2)	81.85	81.17
8	5 (−1)	30 (−1)	2 (−1)	60 (−1)	34.55	35.25
9	10 (+1)	30 (−1)	2 (−1)	60 (−1)	47.25	46.91
10	10 (+1)	30 (−1)	4 (+1)	60 (−1)	70.44	69.95
11	5 (−1)	30 (−1)	2 (−1)	120 (+1)	61.85	61.79
12	7.5 (0)	50 (0)	1 (−2)	90 (0)	28.72	28.18
13	10 (+1)	70 (+1)	4 (+1)	120 (+1)	85.05	84.34
14	7.5 (0)	50 (0)	3 (0)	90 (0)	49.75	49.82
15	10 (+1)	30 (−1)	2 (−1)	120 (+1)	75.56	76.07
16	10 (+1)	30 (−1)	4 (+1)	120 (+1)	94.25	95.04
17	5 (−1)	70 (+1)	2 (−1)	60 (−1)	12.65	11.98
18	5 (−1)	30 (−1)	4 (+1)	60 (−1)	64.04	63.86
19	7.5 (0)	90 (+2)	3 (0)	90 (0)	41.15	41.31
20	10 (+1)	70 (+1)	4 (+1)	60 (−1)	54.88	55.05
21	7.5 (0)	50 (0)	3 (0)	90 (0)	50.55	49.82
22	7.5 (0)	50 (0)	3 (0)	90 (0)	49.75	49.82
23	7.5 (0)	50 (0)	5 (+2)	90 (0)	79.38	79.82
24	12.5 (+2)	50 (0)	3 (0)	90 (0)	80.62	80.42
25	7.5 (0)	10 (−2)	3 (0)	90 (0)	75.55	75.28
26	7.5 (0)	50 (0)	3 (0)	90 (0)	49.35	49.82
27	7.5 (0)	50 (0)	3 (0)	30 (−2)	24.75	25.33
28	5 (−1)	70 (+1)	2 (−1)	120 (+1)	42.25	42.72
29	10 (+1)	70 (+1)	2 (−1)	60 (−1)	27.68	27.96
30	5 (−1)	70 (+1)	4 (+1)	120 (+1)	70.85	71.31

## Resulta and Disscusion

### Analysis of variance (ANOVA)

The results of the analysis of variance test is summarized in Table 4. The probability > F for the model is less than 0.05 which implies that the model is significant and the terms in the model have significant effects on the response. In this case A, B, C, D, AB, AC, AD, BC, BD, CD, A2, B^2^, C^2^, D^2^ are significant model terms at the 95 % confidence level (α =5 %). The model F-value of 1387.59 and *P*-value of < 0.0001 implies that the model is highly significant. Based on the ANOVA results, the values of R^2^, Adjusted R^2^ and Predicted R^2^ were 0.9992, 0.9985 and 0.9971, respectively. This result suggests that the regression model is well interpreted the relationship between the independent variables and the response. Furthermore, the adequate precision ratio of 149.08 in the study shows that this model could be applied to navigate the design space defined by the CCD.

**Table 4:** ANOVA results for Response Surface Quadratic Model

Source	Sum of squares	df	Mean square	F -Value	*P*- value
model	12060.48	1	861.46	1387.59	<0.0001
A-pH	914.15	1	914.15	1472.45	<0.0001
B-TCcon.	1730.94	1	1730.94	2788.08	<0.0001
C-PScon.	3999	1	3999	6441.32	<0.0001
D-Time	4676.04	1	4676.04	7531.85	<0.0001
AB	18.66	1	18.66	30.06	<0.0001
AC	30.97	1	30.97	49.88	<0.0001
AD	6.84	1	6.84	11.01	0.0047
BC	16.4	1	16.4	26.42	0.0001
BD	17.64	1	17.64	28.41	<0.0001
CD	16.61	1	16.61	26.75	0.0001
A^2^	571.64	1	571.64	920.76	<0.0001
B^2^	123.3	1	123.3	198.6	<0.0001
C^2^	29.96	1	29.96	48.26	<0.0001
D^2^	20.18	1	20.18	32.5	<0.0001
Residual	9.31	15	0.62		
Lack of Fit	5.13	10	0.51	0.61	0.7607
Pure Error	4.18	5	0.84		
Cor Total	12069.8	29			

R^2^ = 0.9992; Adjusted R^2^ = 0.9985 and Predicted R^2^ = 0.9971

### Evaluation of model adequacy

There are many statistical techniques for the evaluation of model adequacy, but graphical residual analysis is the primary statistical method for assessment of model adequacy [59].

**Fig 2:**
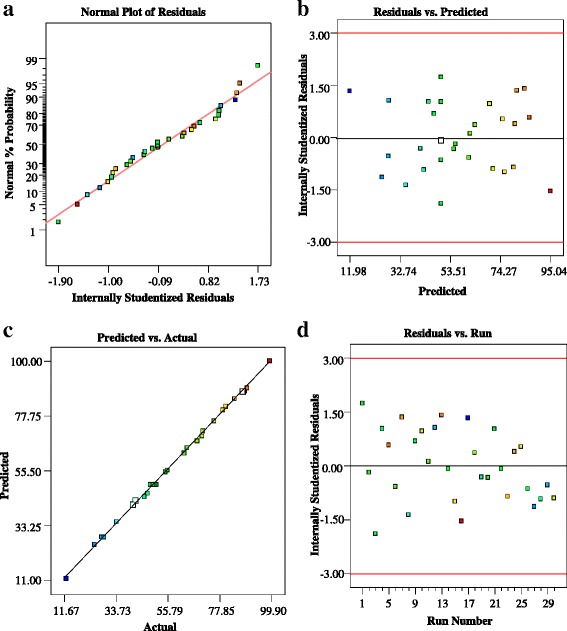
The plot of: (**a**) Normal Plot of Residuals; (**b**) residuals vs Predicted Response; (**c**) predicted vs Actual values; (**d**) Residuals vs Run Order

The normal probability plot indicates that the points on this plot are formed a nearly linear pattern (Fig. 2 (a)). Therefore, the normal distribution is a good model for this data set. Random scattering of the points of internally studentized residual (the residual divided by the estimated standard deviation of that residual) versus predicted values between −3 and +3 emphasizes highly accurate prediction of the experimental data through the derived quadratic model (Fig. 2 (b)).

**Fig 3:**
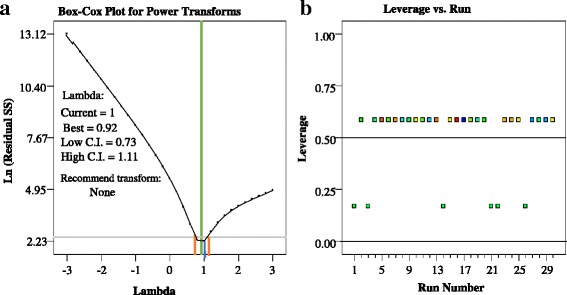
The plot of: (**a**) Box-Cox Plot for Power Transforms; (**b**) The points Leverage vs Run order for the CCD Design

The plot of predicted vs Actual values (Fig. 2 (c)) indicate a higher correlation and low differences between actual and predicted values. Hence, the predictions of the experimental data by developed quadratic models for the TC degradation is perfectly acceptable and this model fits the data better. Also, the random spread of the residuals across the range of the data between −3 and +3 implies that there are no evident drift in this process and the model was a goodness fit (Fig. 2 (d)). The Box-cox plot is used for determine the suitability of a power low transformation for the selected data (Fig. 3 (a)). In this study, the best lambda values of 0.92 was obtained with low and high confidence interval 0.73 and 1.11, respectively. Therefore, recommend the standard transformation by the software is 'None'. The plot of points Leverage vs Run order is shown in Fig. 3 (b). The factorial and axial points have the most influence with a leverage of approximately 0.59, while the center points have the least effect with a leverage of 0.16.

### Design matrix evaluation for response surface quadratic model

Design matrix evaluation implies that there are no aliases for the quadratic model. In general, a minimum of degrees of freedom 3 and 4 has been recommended for lack-of-fit and pure error, respectively. Therefore, degrees of freedom obtained in this study ensured a valid lack of fit test (Table 5).

**Table 5:** Degrees of freedom for evaluation

Model	14
Residuals	15
Lack of Fit	10
Pure Error	5

The standard error (SE) used to measure the precision of the estimate of the coefficient. The smaller standard error implies the more accurate the estimate. The variables of A, B, C and D have a standard errors = 0.16. The interceptions of AB, AC, AD, BC, BD and CD have slightly high standard errors **=** 0.2, while A^2^, B^2^, C^2^ and D^2^ have standard errors = 0.15. An approximate 95 % confidence interval for the coefficient is given by the estimate plus and minus 2 times the standard error. For example, with 95 % confidence can be said that the value of the regression coefficient A is between 6.49 and 5.85 (6.17 ± 2 × 0.16).

The quadratic model coefficients for the CCD are shown in Table 6. This results suggested that the variables coefficients and their interactions are estimated adequately without multicollinearity. The low Ri-squared for independent variables and their interactions imply that the model is a good fit. In general, power should be approximately 80 % for detecting an effect [60]. In this study, there are more than 99 % chance of detecting a main effect while it is twice the background sigma.

**Table 6:** The Quadratic model coefficients for the CCD

Term	StdErr**	VIF	Ri-Squared	Power at 5 %	Power at 5 %	Power at 5 %
				SN = 0.5	SN = 1	SN = 2
A	0.16	1	0	20.90 %	63.00 %	99.50 %
B	0.16	1	0	20.90 %	63.00 %	99.50 %
C	0.16	1	0	20.90 %	63.00 %	99.50 %
D	0.16	1	0	20.90 %	63.00 %	99.50 %
AB	0.2	1	0	15.50 %	46.50 %	96.20 %
AC	0.2	1	0	15.50 %	46.50 %	96.20 %
AD	0.2	1	0	15.50 %	46.50 %	96.20 %
BC	0.2	1	0	15.50 %	46.50 %	96.20 %
BD	0.2	1	0	15.50 %	46.50 %	96.20 %
CD	0.2	1	0	15.50 %	46.50 %	96.20 %
A^2^	0.15	1.05	0.0476	68.70 %	99.80 %	99.90 %
B^2^	0.15	1.05	0.0476	68.70 %	99.980 %	99.90 %
C^2^	0.15	1.05	0.0476	68.70 %	99.80 %	99.90 %
D^2^	0.15	1.05	0.0476	68.70 %	99.80 %	99.90 %

**Basis Std. Dev. = 1.0

### Final equation and model graphs

The values of regression coefficients were determined and the experimental results of CCD were fitted with second order polynomial equation. The quadratic model for TC degradation rate in terms of coded were determined using as following Eq. (9):

### Final equation in terms of coded factors

9 $$ Y= + 49.82+6.17*A-8.49*B+12.91*C+13.96*D+1.08*A*B-1.39*A*C+0.65*A*D+1.01*B*C+1.05*B*D-1.02*C*D+4.57*{A}^2+2.12*{B}^2+1\ .05*{C}^2+0.86*{D}^2 $$

**Fig 4:**
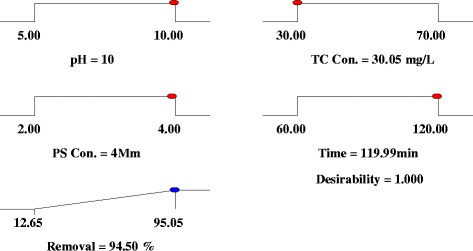
Ramp function graph for the numerical optimization of TC degradation

The factors in the quadratic equation were coded to produce the response surface with limiting the responses into a range of −1 to +1. The ramp function graph for the maximum TC degradation rate is shown in Fig. 4. The optimization of experimental conditions was conducted for maximize the TC degradation at defined criteria of the variable. The developed quadratic model for the TC degradation (Eq. (8)) was applied as an objective function to the optimization of operating conditions. Consequently, the optimum parameters were achieved using the numerical technology based on the predicted model and the variable in their critical range. The maximum degradation of 95.01 % was achieved at pH = 9.9, TC concentration = 30.19 mg/L, PS concentration = 3.97 mM and reaction time = 119.98 min. in order to evaluation of the model validity, the experiments were carried out under the optimal operating conditions. 93.45 % TC degradation was obtained under the optimum operating conditions, which supported the results of the developed model.

**Fig 5:**
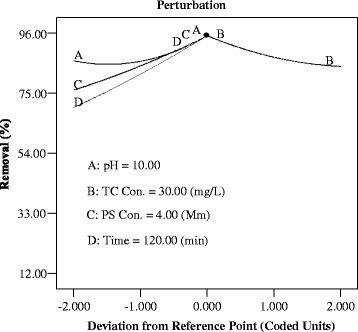
The perturbation Plot of independent variables

The perturbation Plot of independent variables implies that reaction time (D) has the most significant effect (steepest slope) on the TC degradation rate, followed by S_2_O_8_^−2^ concentration (C) and TC concentration (B), whereas pH (A) has the lowest effect on the TC degradation. (Fig. 5).

### Interactive effect of independent variables on the TC degradation

**Fig 6:**
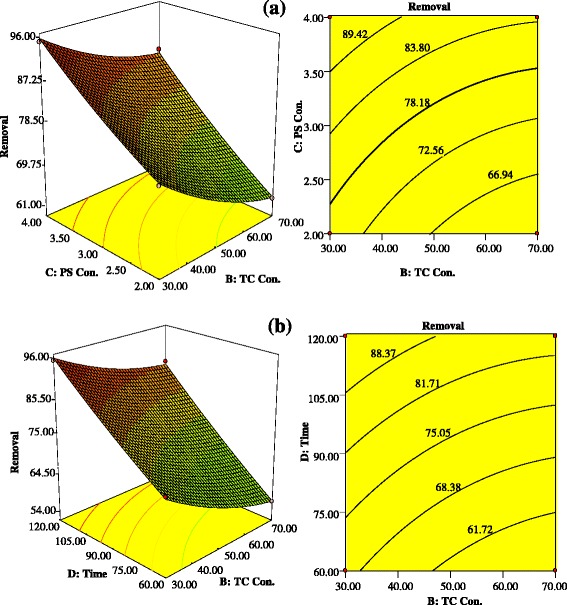
Contour and 3-D plots showing Interactive effect of: (**a**) TC concentration (mg/L) and PS concentration (mM); (**b**) TC concentration (mg/L) and sonication time (min)

**Fig 7:**
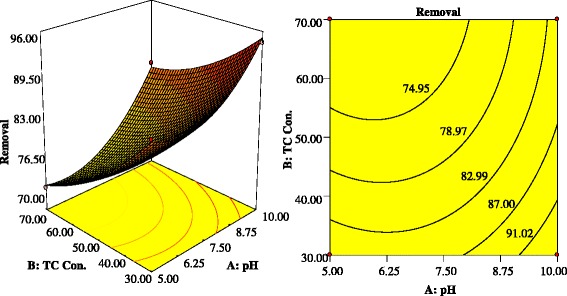
Contour and 3-D plots showing Interactive effect of pH and TC concentration (mg/L)

Three-dimensional surfaces and contour plots are graphical representation of regression equation for the optimization of reaction Status. The results of the interactions between four independent variables and dependent variable are indicated in Figs. 6 and 7.

Figure 6(a) indicates the interaction effect of TC concentration and PS concentration on the TC degradation rate with reaction time of 120 min. with the increasing PS concentration, the TC degradation rate significantly enhanced. With increasing PS concentration from 2 to 4 mM, the TC degradation rate increased from 75.56 % to 94.25 % at TC concentration of 30 mg/L. These results suggest that with increasing PS concentration, more sulfate radicals are produced which leads to more quickly TC degradation [32].

Figure 6(b) indicates the interaction effect of initial TC concentration and reaction time on the TC degradation rate. The TC degradation rate strongly increased with increase of sonication time from 60 to 120 min. with increasing reaction time from 60 to 120 min, TC concentration of 30 and 70 mg/L, the TC degradation rate increased from 70.44 % to 94.25 % at TC concentration of 30 mg/L. With increasing the TC concentration from 30 to 70 mg/L, the TC degradation rate decreased from 94.25 % to 85.05 %. In the constant conditions, with the increasing TC concentration, possibility of reaction between TC molecules and reactive species were declined. Moreover, the higher concentration of TC may lead to the creation of resistant byproducts and consequently decreases the degradation rate of TC [14, 61]. However, the total amount of degraded TC increased with the increasing initial TC concentration. This results are in agreement with the results obtained by other researchers [50].

Figure 7 indicates the interaction influence of pH value and initial TC concentration on the TC degradation rate. With increasing pH from acidic (5) to natural (7.5), the degradation rate slightly decreased, whereas with increasing pH from neutral (7.5) to alkaline (10), the degradation rate significantly enhanced. The TC degradation rate increased from 86.62 % to 94.25 % with increasing pH from 5 to 10, at TC concentrations of 30 mg/L. Under alkaline conditions (pH ≥10), alkaline-activated persulfate is the primary responsible for the production of SO4^**-•**^, O_2_^**-•**^ and HO^**•**^ radicals as following equations: [62, 63].

10 $$ {\mathrm{S}}_2{\mathrm{O}}_8^{2-}+2{\mathrm{H}}_2\mathrm{O}\ \overset{{\mathrm{O}\mathrm{H}}^{-}}{\to }\ {\mathrm{H}\mathrm{O}}_2^{-}+2{\mathrm{S}\mathrm{O}}_4^{2-}+3{\mathrm{H}}^{+} $$

11 $$ {\mathrm{H}\mathrm{O}}_2^{-}+{\mathrm{S}}_2{\mathrm{O}}_8^{2-}\to {\mathrm{S}\mathrm{O}}_4^{-\bullet }+\kern0.5em {\mathrm{S}\mathrm{O}}_4^{2-}+{\mathrm{H}}^{+}+{\mathrm{O}}_2^{-\bullet } $$

12 $$ {\mathrm{SO}}_4^{-\bullet } + {\mathrm{OH}}^{-}\to\ {\mathrm{SO}}_4^{2-} + {\mathrm{HO}}^{\bullet } $$

Also, at alkaline pH, sulfate radicals can react with hydroxyl anions to generate hydroxyl radicals (HO^•^) according to Eq. (3). In addition, a theory was introduced by other researchers that with increasing pH, the PS degradation into HO^•^ and SO_4_^**-**•^ increased [64].

The SO_4_^**-•**^ is the predominant radical responsible for TC degradation at acidic pH, whereas both SO_4_^**-•**^and OH^**•**^ are contributing in TC degradation at natural pH. Thus, three reactions compete with each other in natural pH: the reaction between SO_4_^**-•**^ and HO^**•**^, the reaction between SO_4_^**-•**^ and TC, and the reaction between HO^**•**^ and TC, the simultaneous occurrence of these reactions may reduce the TC degradation rate [37, 65].

### Kinetics of tetracycline degradation

The sonochemical degradation process typically follows pseudo first-order kinetics as shown in the following Eqs. (13) and (14). Many studies have suggested that oxidation of organic pollutants by ultrasound follows pseudo first-order kinetics [42, 47, 52].

13 $$ -\mathrm{d}\left[\mathrm{T}\mathrm{C}\right]/\mathrm{d}\mathrm{t} = \mathrm{k}\left[\mathrm{T}\mathrm{C}\right] $$

Eq. (13) can be rewritten as:

14 $$ \ln\ \mathrm{Ci}/\mathrm{C}\mathrm{t} = \mathrm{k}\mathrm{t} $$

Where C_i_ is the initial TC concentration, C_t_ is the TC concentration at time t, k is the pseudo first order reaction rate t is constant (min^−1^) and the reaction time (min). To study the TC degradation by US/S_2_O_8_^2−^ process, the data obtained was investigated using the pseudo first order kinetics. The effect of different parameters such as initial TC concentration, initial PS concentration, pH and temperature on the kinetic of TC degradation was evaluated. In all the experiments, TC degradation well-fitted to the using the pseudo first order kinetics with higher correlation coefficients (R^2^). The values of kinetic rate constants (k) related to the different parameters, with their regression coefficients R^2^ are shown in Table 7.

**Table 7:** Effect of operation parameters on the kinetics degradation of TC

parameter	Value	*k*_*0*_ (min^−1^) × 10^−2^	R^2^	t _1/2_ (min)
TC concentration (mg/L)	25	2.29	0.9973	30.2
	50	1.75	0.9952	39.6
	75	1.23	0.9956	56.3
PS concentration (mM)	2	1.15	0.9816	60.6
	3	1.52	0.9946	45.9
	4	2.29	0.9973	30.2
pH	5	1.62	0.9937	42.7
	7.5	1.12	0.9942	62.8
	10	2.29	0.9973	30.2
Temperature (°C)	25	2.29	0.9973	30.2
	45	5.70	0.9127	12.1
	55	7.87	0.921	8.8
	65	10.42	0.9824	6.6

### The effect of temperature on the degradation of tetracycline

To investigate the effect of temperature on the TC degradation rate, experiments were done with various temperature varying from 25 to 65 °C. With increasing temperature from 25 to 65 °C, the degradation rate constant increased from 0.0229 to 0.1042 min ^−1^. Complete TC degradation occurs after 40, 60 and 75 min of reaction at 65, 55 and 45 °C respectively. The activation of S_2_O_8_^2−^ can be done under heat to form SO_4_^**-•**^ radical as following Eq. (15). Therefore, complete removal of TC by high temperature could be as a result of thermally activated S_2_O_8_^2−^ oxidation. Moreover, the increase of temperature significantly enhanced the cavitation activity and chemical effects, resulting in greater degradation rate of TC by US/S_2_O_8_^2−^ process [22, 60].

15 $$ {\mathrm{S}}_2{\mathrm{O}}_8^{-2}+\overset{\mathrm{Termal}-\mathrm{activation}}{\to }2{\mathrm{S}\mathrm{O}}_4^{-\bullet}\kern4em \left(30{}^{\circ}\mathrm{C}<\mathrm{T}<99{}^{\circ}\mathrm{C}\right) $$

To investigate the effect of ultrasound on the process kinetics, significant parameters such as activation energy (Ea) play a remarkable role. The effect of temperature on the rate of the reaction and rate constant (k) is obtained by Arrhenius equation according with Eq. (16) [66].

16 $$ \mathrm{L}\mathrm{n}\mathrm{K}=\mathrm{A}\  \exp\ \left(-\frac{\mathrm{Ea}}{\mathrm{RT}}\ \right) $$

**Fig 8:**
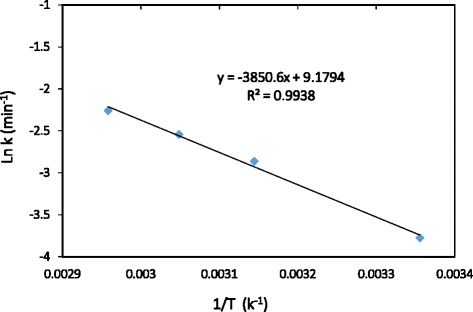
Arrhenius equation graph representation the temperature dependence on chemical reaction rate

Arrhenius plot can be used to calculate the Activation Energy at various temperatures by graphing ln k (rate constant) versus 1/T (kelvin). The graph between ln k and 1/T is a straight line with an intercept of ln A and the slop of the graph is equal to –E_a_/R, where R is a constant equal to 8.314 J/mol-K. According with Arrhenius plot (Fig. 8), the activation energy values of 32.01 (kJ/mol) obtained for degradation of TC by S_2_O_8_^2−^/US process. It means that for a successful reaction, the colliding molecules must have a total kinetic energy of 32.01 kJ/mol. The low activation energy indicates that the degradation of TC by S_2_O_8_^2−^/US process is thermodynamically feasible.

### Mineralization, Changes of ultraviolet Visible (UV–Vis) spectra and the proposed degradation pathway

**Fig 9:**
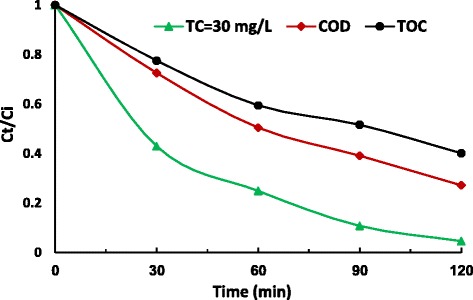
Removal of TC, COD and TOC by US/S_2_O_8_^2-^ process; [S_2_O_8_^2-^] = 4 mM; US: 500 W, 35 KHz; pH=10; T=25 ^0^C

Usually, sonochemical treatment lead to degradation of structure and ultimately mineralization of organic compounds [67]. While perfect mineralization for most antibiotics are difficult because of the structural stability [68]. Therefore, the changes of TOC and COD were evaluated during US/S_2_O8^2−^ process and the result are shown in Fig. 9. After 120 min of reaction, TC, COD and TOC were removed approximate 95 %, 73 % and 60 %. The incomplete mineralization implies the potential formation of intermediate products and further identification of the degradation by-products is required.

**Fig 10:**
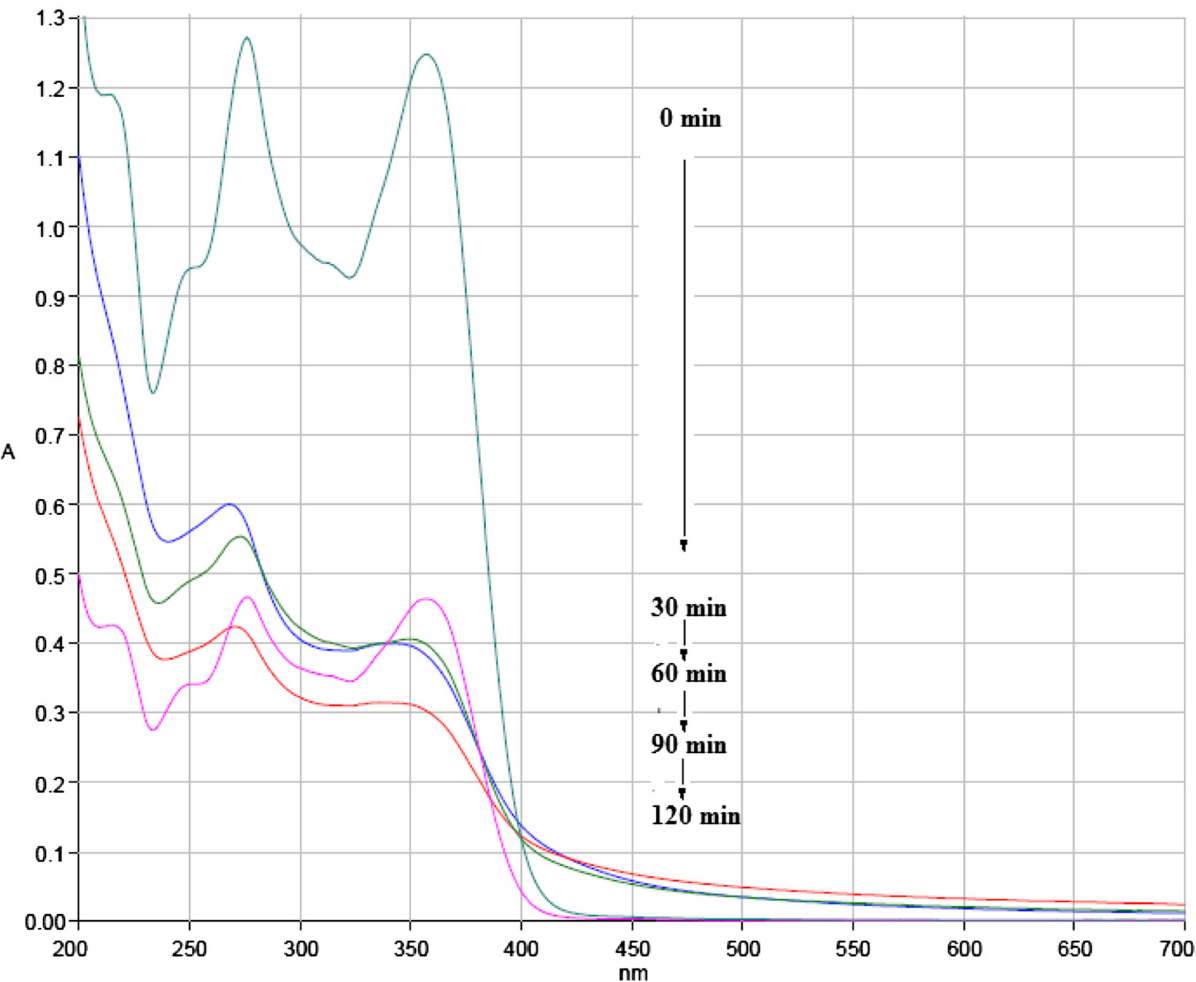
Changes of UV–Vis spectra of 50 mg/L aqueous solution of TC during process of US/S_2_O_8_^2-^

**Fig 11:**
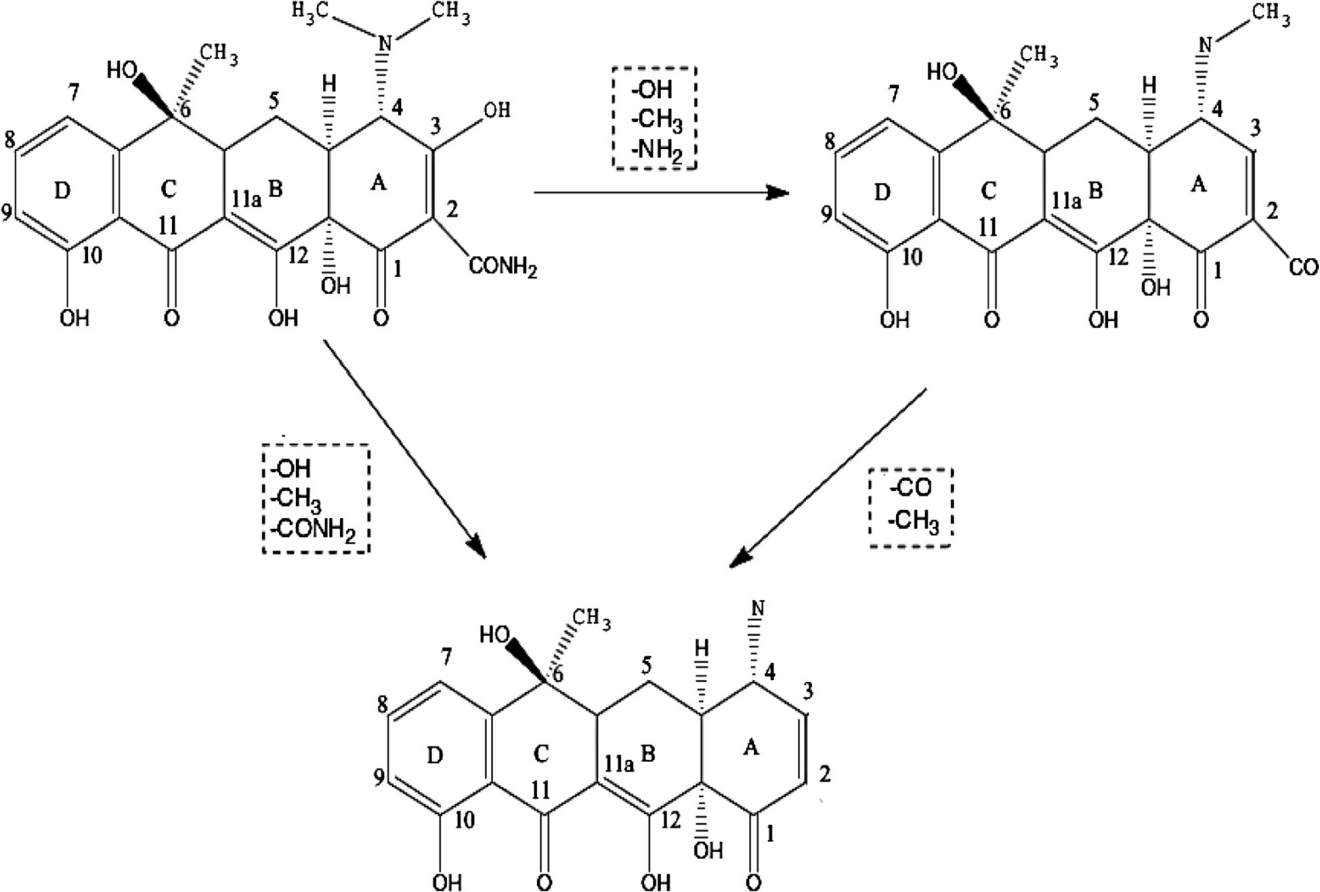
The proposed degradation pathway for tetracycline S_2_O_8_^2−^

**Fig 12:**
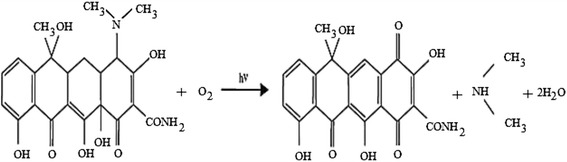
Photodegradation of TC into 4a, 12a-anhydro-4-oxo-4-dedimethylaminotetracycline

To evaluate structural changes of TC, the UV–Vis spectra obtained before and after US/ S_2_O_8_^2−^ process in various time are shown in Fig. 10. The UV–Vis spectra obtained before process indicates two main absorption bands at 275 and 360 nm. The absorption of TC in 360 nm is due to aromatic rings B–D, such as the developed chromophores [68, 69]. With increase of reaction time, the absorption band slightly decreased because of the fragmentation of phenolic groups attached to aromatic ring B [69, 70]. The generation of acylamino and hydroxyl groups led to reduction of absorbance at 270 nm band [70]. The absorption decay at 360 nm band faster than 275 nm. This implies that the ring containing the N-groups (responsible for the absorbance at 276 nm) hardly opened than the other rings, or the created intermediate products absorbed at this wavelength [71]. The proposed degradation pathway for tetracycline based on loses of N-methyl, hydroxyl, and amino groups is shown in Fig. 11. This possible pathway corresponded with conducted studies by other researchers [72]. In addition, TC has a naphthol ring with high stability, which remains unchanged in the reaction and is not easily mineralized. Also, the absorption decay at 360 nm band was found with a relatively small absorption in the visible region. This could be due to the forming of 4a,12a-anhydro-4-oxo-4-dedimethylaminotetracycline according to Fig. 12 [73].

### Performance evaluation of US/S_2_O_8_^2−^ process in the removal of different organics

An overview of performance of US/S_2_O_8_^2−^ process for removal of different organics along with present study was presented in Table 8. The overview confirm that US/S_2_O_8_^2−^ process is an attractive alternative technique for degradation of the wide range of organic compounds in aqueous solutions. This process could effectively decomposed organic pollutants in aqueous solution, and the degradation rate depends heavily on the operating conditions, such as physical and chemical characteristics and initial concentration of pollutant, S_2_O_8_^2−^ concentration, initial pH, reaction time, ultrasound power, ultrasound frequency, and temperature of the medium. Therefore, the various experimental conditions could lead to the various removal efficiencies of organic compounds using US/S_2_O_8_^2−^ process.

**Table 8:** Degradation of different types of organic pollutants in aqueous solutions using US/S_2_O_8_^2−^ process

compound	Concentration (mg/L)	operating conditions	Summary of results	reference
Tetracycline	100	[S_2_O_8_^2−^] = 200 mM; US = 80 W, 20 KHz; pH = 3.7; T = ambient	More than 51 % degradation after 120 min.	Hou et al. [48]
Trichloroethane	50	[S_2_O_8_^2−^] = 0.94 mM US = 400 kHz, 100 W pH = 7; T = 20 ± 2 °C	100 % degradation after 120 min.	Li et al. [42]
Perfluorooctanoic acid	50	[S_2_O_8_^2−^] = 46 mM US = 150 W, 40 KHz pH = 4.3: T = 25 °C	More than 98 % degradation after 120 min.	Lin et al. [52]
2,4 Dichlorophenol	50	[S_2_O_8_^2−^] = 4 Mm US = 40 KHz, pH = 3; T = 30 °C	More than 95 % degradation after 60 min.	Seid Mohammai [74]
Acid Orange 7	30	[S_2_O_8_^2−^] = 1.25 Mm US = 100 W, 20 kHz pH = 5.8; T = ambient	More than 10 % degradation after 20 min.	Wang et al. [75]
Tetracycline	30	[S_2_O_8_^2−^] = 4 Mm US = 500 W, 35 KHz pH = 10; T = 25 ± 2 °C	More than 95 % degradation, COD and TOC removal of 72 % and 59 % after 120 min	Present study

## Conclusion

Sonochemical degradation of TC in the presence of S_2_O_8_^2−^ was investigated with focusing on the optimizing of the operation parameters such as pH, S_2_O_8_^2−^ concentration, initial TC concentration and reaction time. This study indicated that RSM was the suitable method to optimizing the best operating conditions to maximizing the TC degradation. The reaction time showed the highest effect on the TC degradation, followed by initial S_2_O_8_^2−^ concentration, initial TC concentration and pH. Under optimal conditions, the TC degradation rate, COD and TOC removal efficiency were found to be 95.01 %, 72.8 % and 59.7 %, respectively. The degradation process followed the pseudo-first-order kinetics. The activation energy value of 31.71 kJ/mol implies that the degradation of TC by US/S_2_O_8_^2−^ process is thermodynamically feasible. The ultraviolet visible spectra obtained before and after ultrasound irradiation in the presence of S_2_O_8_^2−^ indicated that proposed degradation pathway for tetracycline was based on loses of N-methyl, hydroxyl, and amino groups. Overall, US/S_2_O_8_^2−^ process was found to be a promising technology for TC degradation in aqueous solution.
